# Bevacizumab Inhibits Angiogenic Cytokines in Head and Neck Squamous Cell Carcinoma: From Gene to the Protein

**Published:** 2018-04-01

**Authors:** Hossein Heydar, Kamran Mansouri, Maryam Norooznezhad, Fatemeh Norooznezhad, Abdolreza Mohamadnia, Naghmeh Bahrami

**Affiliations:** 1 Craniomaxillofacial Research Center, Tehran University of Medical Sciences, Oral and Maxillofacial Surgery Department, School of Dentistry, Tehran University of Medical Sciences, Tehran, Iran; 2 Medical Biology Research Center, Kermanshah University of Medical Sciences, Kermanshah, Iran; 3 Virology Research Center, National Research Institute of Tuberculosis and Lung Diseases (NRITLD), Shahid Beheshti University of Medical Sciences, Tehran, Iran; 4 Department of Biotechnology, School of Advanced Technologies in Medicine, Shahid Beheshti University of Medical Sciences, Tehran, Iran

**Keywords:** Head and neck squamous cell carcinoma, Bevacizumab, Angiogenesis, Vascular endothelial growth, Matrix metalloproteases

## Abstract

**Background: **Head and neck squamous cell carcinoma (HNSCC) is one most prevalent cancers among worldwide. Aim of this study was to evaluate possible effect of bevacizumab, a vascular endothelial growth (VEGF) factor monoclonal antibody on HNSCC cells in vitro to evaluate angiogenic profile changes.

**Materials and Methods: **HNSCC cells were grown and after that different concentrations of bevacizumab were added in order to evaluate cytotoxic concentration using MTT assay. Then after, the cultured cells in presence of different concentration of bevacizumab were evaluated for gene expression of VEGF, matrix metalloprotease-2 (MMP-2) and MMP-9 using real time polymerase chain reaction (PCR). Moreover, the VEGF expression was evaluated by enzyme-linked immunosorbent assay (ELISA).

**Results: **The concentration at which half cells died (IC59) was calculated 1779 µg/mL and at this concentration, VEGF protein secretion was decreased by over one fold. RT-PCR results showed that MMP2, MMP9 and VEGF decreased by 1, 0.6 and 1.1 folds, respectively.

**Conclusion:** It seems that bevacizumab could be considered as a side therapy for patients with HNSCC due to its potential for inhibition of angiogenic related factors, but further complementary studies are necessary.

## Introduction

 Head and neck squamous cell carcinoma (HNSCC) as the sixth most prevalent cancer type worldwide consists of about 5% of all cancers diagnosed annually in the United States ^   1 ^ and unfortunately, 50% of HNSCC patients develop recurrent or metastatic (R/M) SCCHN with poor prognosis ^   2 ^. In 1971, Folkman proposed that the growth of solid neoplasms is always accompanied by neovascularization ^3^^, ^^4^. Later it was found that anti-angiogenesis agents might be an effective therapeutic strategy to treat cancers ^   5 ^ or other angiogenic-related diseases such as corneal neovascularization, psoriasis, and hemophilic arthropathy ^6^^-^^8^. In 1980s, a key regulating factor for blood vessel growth, under both physiologic and pathologic conditions, was discovered and named vascular endothelial growth factor (VEGF). In 1993, a monoclonal antibody was introduced to address VEGF in order to block it and therefore suppress tumor growth in vivo, which was later humanized and led to the development of bevacizumab (Avastin^®^) ^   9 ^. General receptors for VEGF have been reported as VEGFR-1 (Flt-1) and VEGFR-2 (Flk-1) both expressed on EC surface. Matrix metalloproteases (MMPs) are among other agents involved in angiogenesis to facilitate ECs migration and invasion by digesting extracellular matrix. These migrating ECs would further multiply under the influence of VEGF and other tumor-derived growth factors ^   10 ^. Bevacizumab is approved by FDA and is successfully used in many types of cancers either alone or in combination with other chemotherapy drugs and radiotherapy. In a study done on colorectal cancer, bevacizumab plus fluorouracil-based chemotherapy was found to be a good option for the treatment of metastatic colorectal cancer ^   11 ^. In a phase III investigation, Bevacizumab was used for patients with recurrent cervical cancer as the second- and third-line of treatment with a quit satisfying response ^   12 ^. Moreover, mouse models of HNSCC treated with bevacizumab/paclitaxel combination exhibited greater angiogenesis inhibition than either agent alone and apoptosis was also induced in tumor cells ^   13 ^. Altogether, this study aimed to investigate the possible anti-proliferatory and anti-angiogenic effects of bevacizumab on head and neck cancerous cell line (HN5) and human umbilical vein endothelium cells (HUVEC), respectively.

## MATERIALS AND METHODS

**Reagents**

Dulbecco’s Modified Eagle’s Medium (DMEM) (Gibco, Belgium), Fetal Calf Serum (FCS), and trypsin–EDTA were purchased from Invitrogen (Life Technologies, Gaithersburg, MD, USA), streptomycin and penicillin were purchased from Sigma-Aldrich (Taufkirchen, Germany). Real- time PCR and cDNA synthesis kit were obtained from EUREX (EUREX Co, Poland). Bevacizumab (Avastin) was from Roche (Roche Pharma, Switzerland). VEGF ELISA kit was Bioassay Technology Laboratory Kit (Shanghai Korain Biotech CO.LTD).

**Cell Culture**

HN5 cell line was obtained from Pasteur Institute of Iran and was further cultured in DMEM containing 10% (v/v) heat inactivated FCS and 100 U/mL penicillin and 100µg/mL streptomycin. Cells were incubated at 37 °C in a humidified incubator under an atmosphere of 85% humidity and 5% CO2.

**Cell viability assay**

HN5 cells were cultured into 96-well plates at a density of 2×10^3^ per well in DMEM containing 10 % (v/v) FCS and already mentioned antibiotics at 37°C for 24h in an incubator. 24h later, cell medium was replaced with fresh DMEM containing bevacizumab ranging from 250 to 8000 µg/mL in triplicates. After another 24h, cell viability assay was performed using 20μl of 3-(4, 5-dimethylthiazolyl-2)-2, 5-diphenyltetrazolium bromide (MTT, Sigma, USA) solution added to each well (5 mg/ml in PBS). An incubation of 4h at 37°C was allowed and followed by adding 200µl of DMSO to each already medium-emptied well in order to dissolve purple *formazan crystals*. At last, the absorbance was read at 570 nm, with background subtraction of 630 nm using a plate reader (Space fax 2100, Awareness, USA).

**Enzyme-linked immunosorbent assay (ELISA)**

The HN5 cell line was cultured at the density of 2×10^5^ in 25cm^3^ flasks within DMEM medium supplemented with 10% FBS for 24h at 37 °C, 5% CO_2_ and 85% humidity. Afterwards, the cells were treated with different concentrations of bevacizumab (in serum-free medium), including 0, 500, 1000, 2000, 4000, and 8000 µg/mL for 24h. Then, the cell-free supernatants were collected from each well and the concentration of VEGF secreted by cells was quantified using a VEGF ELISA kit Bioassay Technology Laboratory Kit (Shanghai Korain Biotech CO.LTD) in accordance to manufacturer’s instructions. The sensitivity of the kit was 5pg/ml.

**Real-time polymerase chain reaction (PCR)**

Total RNA extraction was performed by RNAX-Plus (Cinaclon, Iran) (after 18h of incubating HN5 cells were treated with different concentration of bevacizumab, including 0, 500, 1000, 2000, 4000, and 8000 µg/mL at 37 °c, 5% CO2 and 85% humidity) and cDNA synthesis from RNAs was done by EUREX kit according to its instruction. VEGF, MMP-2, 9 mRNA levels were assessed with SYBR Green I and amplified with the Rotor Gene 6000 system (Corbett Research, Australia) by real-time PCR. Beta-actin (β-actin) was used as a reference gene for normalization. The used primers for this method are shown in Table 1.

**Table 1:** Primers used for real-time PCR

Gene	Sense	Anti-sense
β-actin	5´-CTACAATGAGCTGCGTGTGG-3´	5´-AGCTCTTCTCCAGGGAGGA-3´
VEGF	5´-GGCTGGCAACATAACAGAGAA-3´	5´-CCCCACATCTATACACACCTCC-3´
MMP-2	5´-CAGGCTCTTCTCCTTTCACAAC-3´	5´-AAGCCACGGCTTGGTTTTCCTC-3´
MMP-9	5´-TGGGCTACGTGACCTATGACAT-3´	5´-GCCCAGCCCACCTCCACTCCTC-3´

**Data analysis**

The obtained data were analyzed using GraphPad Prism^®^ software as well as graph designing. Statistical significance of all experimental data was determined using two- way analysis of variance (ANOVA) and a P-value < 0.05 was considered to be statistically significant.

## Results

**Cell Viability**

**Fig 1:**
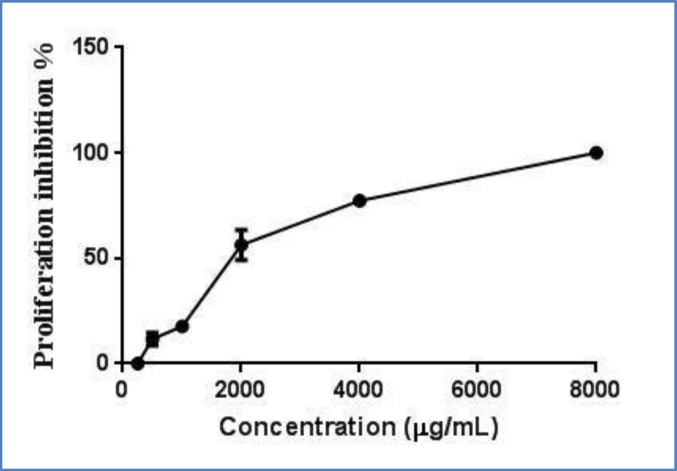
Proliferation inhibition of HN5 cells treated with different concentrations of bevacizumab ranged from 0 (control)- 8000 µg/mL . IC50 was calculated 1779 µg/mL

After 48 h treatment of HN5 with different concentrations of bevacizumuab ranging from 0 (as control) to 8000 µg/mL, cell viability was assessed through MTT assay. Results showed a dose-dependent influence on cell viability. As it is clear from the proliferation chart presented in Figure 1, proliferation was decreased in a dose-dependent manner upon increasing bevacizumuab. The IC50 was calculated to be 1779 µg/mL. 

**ELISA assay**

**Fig 2:**
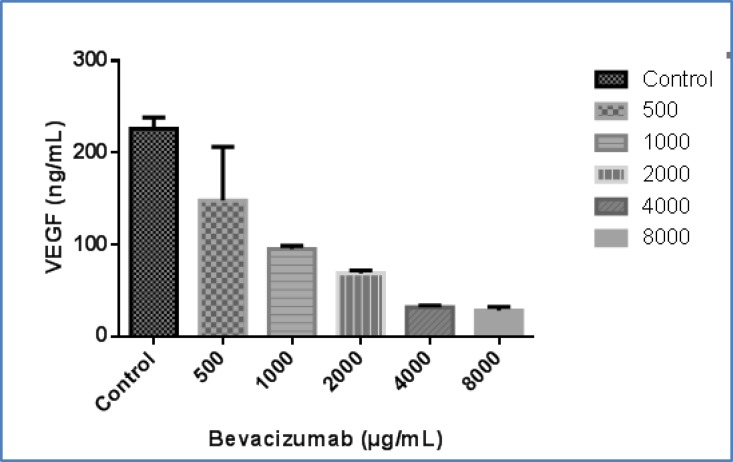
The effect of bevacizumab at concentrations of 0 (as control)-8000µg/mL on secretion of VEGF protein from HN5 cells which shows a reduced pattern upon bevacizumab concentration. VEGF is decreased by over one fold at 2000 µg/mL

Regarding the importance of VEGF in angiogenesis, the inhibitory effects of bevacuzumab on VEGF protein secretion by HN5 cell line was assessed. As illustrated in Figure 2, treating the cells with this monoclonal antibody at the concentrations of 0 (as control), 500, 1000, 2000, 4000, and 8000 µg/mL led to a decreased VEGF secretion pattern in a dose-dependent manner. As perceived from the graph in Figure 2, VEGF is decreased by over one fold at IC50.

**Real-time PCR**

**Fig 3:**
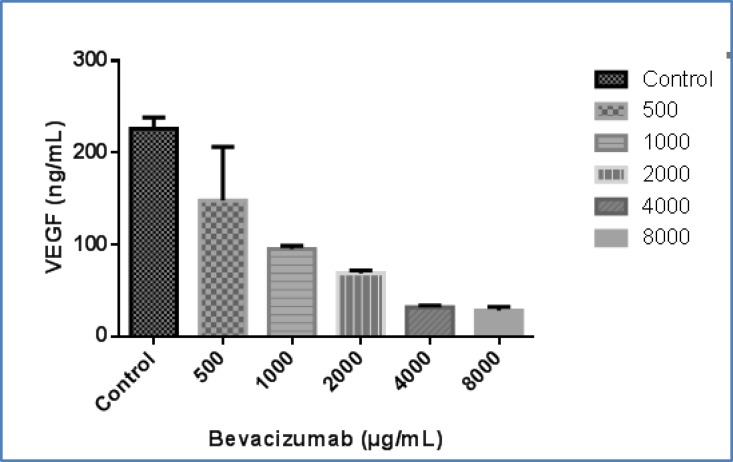
The effect of bevacizumab on the expression of A. MMP2, B. MMP9, and C. VEGF genes in HN5 cell line at the concentration of 2000µg/mL has led to a decrease by 1, 0.6, and 1 folds, respectively

Real-time-PCR approach was applied in order to investigate the inhibitory effect of bevacizumab on VEGF and MMP2,9 expressions. Changes in the ratio of VEGF/MMP2,9 expression to β-actin expression was considered representative for the level of gene expression changes induced. The results illustrated significant downregulation of VEGF expression in HN5 cell line treated with the bevacizumab. As shown in Figure 3, at the concentration of 2000 µg/mL (IC50), MMP2, MMP9, and VEGF gene expressions have been reduced by 1, 0.6 and 1.1 folds, respectively.

## Discussion

 According to the literature tumors only grow less than few millimeters if they don’t access to the new blood supplies by angiogenesis ^   14 ^. In the tumoral hyper-proliferation state, normal intracellular fluid does not meet tumoral needs of nutrition and oxygen, and therefore is not considered for an acceptable resource anymore. Thus, in this situation the balance between angiogenic inducers and inhibitors changes in favor of inducers. Under this condition, the cancer cells start to induce angiogenesis by expression and release of angiogenic growth factors which finally leads to neovascularization and attaining new resources ^   15 ^. Considering this mechanism, different treatment options have been introduced for targeting angiogenesis so far ^16^^, ^^17^. Moreover, targeting angiogenesis has other benefits than only focusing on cancer cells with cytotoxic agents. The most common explanation for this fact is lower rate of resistance to anti-angiogenesis therapies. ^   18 ^ HNSCC, as well as other cancers, has shown an increasing resistance to chemotherapy during the past decades. ^   19 ^ On the other hand, due to the higher genetically stability in endothelial cells, lower resistance to therapies has been seen. Thus, this may be a strength point for anti-angiogenesis therapeutic agents.

So far, bevacizumab has been used in combination with chemoradiation in HNSCC patients with satisfactory results. As Nyflot et al. stated, using this combination led to an increase of 61.3 months in patients lifetime with stage IV of HNSCC ^   20 ^.

As mentioned before, the aim of this study was to investigate the possible effect of bevacizumab on angiogenic profile of HNSCC cell lines. Up until now, anti-angiogenic activity of this monoclonal antibody has been shown on endothelial cells ^   9 ^ which has led to compelling basic and clinical results ^   21 ^. Regarding these results, bevacizumab is now approved by Food and Drug Administration of the United States for different malignancies such as metastatic colon cancer, advanced non-squamous non-small cell lung cancer (NSCLC), ovarian cancer, renal cell carcinoma (RCC) and glioblastoma multiforme ^   22 ^. Thus, authors decided to evaluate some of the changes in the most important angiogenic-inducer cytokines after treatment of HNSCC cells with bevacizumab. As mentioned earlier, cancer cells are able to express and release angiogenic growth factors. According to the results, bevacizumab inhibited proliferation of HNSCC with the IC_50 _of 1779 µg/mL. Moreover, this monoclonal antibody was able to suppress VEGF, MMP-2 and MPP-9 gene expression in HNSCCs. Furthermore, after evaluation of gene expression using ELISA, it was shown that VEGF was suppressed on the protein level. Interestingly, results from VEGF section is completely compatible with RT-PCR data obtained on VEGF gene expression. It is noteworthy to mention that at this concentration, ELISA and RT-PCR results converged for VEGF.

According to the literature, VEGF is responsible for proliferation, migration, and survival of endothelial cells ^   23 ^. Also, it induces MPPs expression in endothelial cells ^   24 ^. As mentioned before, MMPs are vital for degradation of basement membrane which finally helps migration of endothelial cells ^   25 ^. 

## CONCLUSION

 This study showed that bevacizumab is able to inhibit VEGF, MMP-2, and MMP-9 expression in HNSCC from gene to the protein. Thus, considering the earned results, it seems that inhibition of angiogenesis through down-regulation of MMP-2, MMP-9, and VEGF in HNSCC is one the possible mechanisms of cancer treatment. At the end, authors of this study suggest complementary studies on other possible pathways of action of bevacizumab on HNSCC.
